# Fungi under Modified Atmosphere—The Effects of CO_2_ Stress on Cell Membranes and Description of New Yeast *Stenotrophomyces fumitolerans* gen. nov., sp. nov.

**DOI:** 10.3390/jof9101031

**Published:** 2023-10-19

**Authors:** David Heidler von Heilborn, Jessica Reinmüller, Andrey Yurkov, Peter Stehle, Ralf Moeller, André Lipski

**Affiliations:** 1 Institute of Nutritional and Food Science, Food Microbiology and Hygiene, University of Bonn, Friedrich-Hirzebruch-Allee 7, 53115 Bonn, Germany; dheidler@uni-bonn.de (D.H.v.H.);; 2 Leibniz Institute DSMZ—German Collection of Microorganisms and Cell Cultures, Department of Bioresources for Bioeconomy and Health Research, Inhoffenstraße 7 B, 38124 Braunschweig, Germany; andrey.yurkov@dsmz.de; 3 Institute of Nutritional and Food Science, Nutritional Physiology, University of Bonn, Nussallee 9, 53115 Bonn, Germany; p.stehle@uni-bonn.de; 4 Aerospace Microbiology Research Group, Institute of Aerospace Medicine, German Aerospace Center, 51147 Cologne, Germany; ralf.moeller@dlr.de

**Keywords:** carbon dioxide, modified atmosphere packaging, fatty acids, membrane fluidity, filamentous fungi, novel yeast species, capnophiles

## Abstract

High levels of carbon dioxide are known to inhibit the growth of microorganisms. A total of twenty strains of filamentous fungi and yeasts were isolated from habitats with enriched carbon dioxide concentration. Most strains were derived from modified atmosphere packed (MAP) food products or mofettes and were cultivated under an atmosphere of 20% CO_2_ and 80% O_2_. The influence of CO_2_ on fungal cell membrane fatty acid profiles was examined in this study. Major changes were the increase in linolenic acid (C_18:3_ cis 9, 12, 15) and, additionally in most strains, linoleic acid (C_18:2_ cis 9, 12) with a maximum of 24.8%, at the expense of oleic (C_18:1_ cis 9), palmitic (C_16:0_), palmitoleic (C_16:1_ cis 9) and stearic acid (C_18:0_). The degree of fatty acid unsaturation increased for all of the strains in the study, which consequently led to lower melting temperatures of the cell membranes after incubation with elevated levels of CO_2_, indicating fluidization of the membrane and a potential membrane malfunction. Growth was reduced in 18 out of 20 strains in laboratory experiments and a change in pigmentation was observed in several strains. Two of the isolated strains, strain WT5 and strain WR1, were found to represent a hitherto undescribed yeast for which the new genus and species *Stenotrophomyces fumitolerans* (MB# 849906) is proposed.

## 1. Introduction

Fungi are indispensable in food science and are not only used as edible mushrooms and in the production process of fermented and baked goods, but for the refinement of important consumer goods, such as for the maturation of cheese and dry-cured meat [1]. In the maturation process of food products, they release secondary metabolites, which subsequently impart characteristic aromas or flavours [1]. On the other hand, several species of filamentous fungi and yeasts are relevant as food-spoilage organisms, which can cause off-odour and off-flavour of the product, and in some cases even result in contamination with mycotoxins [2]. Food products, especially those that are nutrient-rich and have high water content, such as meat, are prone to microbial spoilage due to microbial growth and metabolic activity [3]. Fungi commonly found on food include several yeast species and molds of the genera *Saccharomyces*, *Rhodotorula*, *Aspergillus*, *Penicillium*, *Mucor* and *Rhizopus* and are food spoilage organisms in most of the cases [4,5]. Fungi are ubiquitously distributed and occur in large numbers of up to 10,000 spores in 1 m³ of air [6], which indicates the importance of their role not only in terms of food production, food waste and hygiene, but also in biotechnology, human medicine and plant pathology.

For conservation and for the prevention of microbial growth, food products are packed under modified atmospheres (MA), including high amounts of CO_2_, depending on the product. For raw red meat, gas mixtures of 20–30% CO_2_ and 70–80% O_2_ are commonly used, whereas for white non-processed fish gas mixtures of 30% O_2_, 40% CO_2_ and 30% N_2_ are used [7]. In general, the function of carbon dioxide is mainly the inhibition of microbial growth [7,8]. In the case of fungi, it has also been shown that the production of several mycotoxins, including aflatoxins, patulin and roquefortine C, is greatly decreased by high CO_2_-containing MA [8,9]. Nevertheless, some food spoilage and plant or human pathogenic microorganisms are able to grow under these conditions, including the fungi *Mucor plumbeus*, *Fusarium oxysporum* and members of the genus *Byssochlamys* [8] and the bacteria *Brochothrix thermosphacta*, *Campylobacter jejuni* and members of the *Lactobacillales* [7,10].

Microorganisms, which can tolerate or even require high-CO_2_ concentrations, are referred to as capnotolerant or capnophilic organisms, respectively. They can be found in anthropogenic CO_2_-rich environments, such as MAP food products, as well in natural environments, such as mofettes or geysers [11]. The latter result from volcanic activities releasing CO_2_ to the surface and thereby create natural unique environments with elevated CO_2_ levels, where even new fungal species have been discovered [12]. Fungal adaptation mechanisms to such an increased presence of the carbon dioxide stressor are expected to be similar for strains from artificial (MAP) and natural habitats (mofettes), which can only be evaluated through comparison analysis. Not much is known about the cell effect of elevated CO_2_ concentrations on microorganisms, but it has been suggested to be a complex interaction of several reactions leading to growth reduction of the organisms or even bactericidal and sporicidal effects [13]. Due to its unique property of being lipo- and hydrophilic, carbon dioxide can dissolve in the phospholipid bilayer of the membrane of cells and is supposed to cause the so-called ‘anesthesia effect’, a loss of order by the incorporation of foreign molecules [14].

The lipids of the phospholipid bilayers of fungi include fatty acids, phospholipids, sterols, oxylipins, sphingolipids and glycolipids [15]. In fungi, the most abundant fatty acids in membrane phospholipids, as well as storage triacylglycerols, are palmitic (C_16:0_) and stearic (C_18:0_) acids, as well as their unsaturated derivatives palmitoleic (C_16:1_ cis 9), oleic (C_18:1_ cis 9), linoleic (C_18:2_ cis 9, 12) and alpha- or gamma-linolenic (C_18:3_ cis 9, 12, 15 or C_18:3_ cis 6, 9, 12) acids [16,17,18]. Polar lipids are therefore an essential part of membranes and fungal polar lipids can account for up to 85% of the total lipid content [16]. The main fungal lipids are phospholipids such as phosphatidylcholine and phosphatidylethanolamine, but also phosphatidylserine, phosphatidylinositol, phosphatidylglycerol and diphosphatidylglycerol, as well as glycolipids [19]. Slightly elevated concentrations of about 560 ppm CO_2_ (instead of the ambient atmospheric 360 ppm of that time) have been shown to stimulate fungal activity and increase the total biomass [20]. In other studies, slightly increased CO_2_ levels have led to increased extracellular enzymatic activities and stimulated microbial respiration [21].

In this work, the effect of high CO_2_-containing MA on several fungal isolates and their membranes was examined. The strains were mainly isolated from MAP food and mofettes and identified based on marker gene sequencing. The strains were furthermore analyzed regarding their fatty acid profile changes towards CO_2_ and temperature stress. The study also resulted in the isolation of a novel yeast species, which was characterized and formally described in this study.

## 2. Materials and Methods

### 2.1. Isolation and Ecology

The majority of the organisms used in this study were isolated from food packed under CO_2_-enriched atmospheres. Oxygen and carbon dioxide concentrations were measured using an Oxybaby M+ O_2_/CO_2_ gas analyzer (WITT-Gasetechnik, Witten, Germany). For the isolation of strains, 10 g of each product were taken and diluted with Ringer’s solution (Merck, Darmstadt, Germany) to a total weight of 100 g. The sample was then homogenized for 60 s and a ten-fold dilution series was prepared. The suspension (0.1 mL) of an appropriate dilution was plated on YMG agar with penicillin and streptomycin (5.0 g glucose, 2.0 g yeast extract, 5.0 g malt extract, 10 g bacteriological agar, 0.04 g penicillin G potassium salt, 0.03 g streptomycin sulfate, *ad* 500 mL distilled H_2_O) and incubated at 25 °C. After 4 d, the isolates were picked from the plates.

All strains used in this study are listed in Table 1. Yeast strains WR1, WT2, WT5, WT6 and WT7 were recovered from a vegetarian wrap packed under MA, with remaining oxygen and carbon dioxide concentrations of 0.3% and 28.4%, respectively. Yeast strain HT4 and filamentous fungus strain HR2 were isolated from MAP minced meat, with a remaining atmosphere of 59.4% O_2_ and 28.0% CO_2_. Yeast strain LR1 and mold strain LT1 were recovered from MAP salmon, with a remaining atmosphere of 0% O_2_ and 21.1% CO_2_. The filamentous fungus strain KR3 was recovered from MAP coffee powder, with a remaining atmosphere of 0.8% O_2_ and 81.2% CO_2_.

In search of natural environments with elevated CO_2_ levels, water samples were taken from mofettes, located at lake Laacher See, Germany (coordinates: 50°24′51.3″ N, 7°17′08.9″ E). A ten-fold dilution series was prepared and YMG agar was inoculated with the suspension (0.1 mL) of an appropriate dilution and incubated at 10 °C and 25 °C to isolate mesophilic fungi that grow at colder and ambient temperatures. Colonies were picked after 4 d of incubation. Yeast strains M3 and M12, as well as filamentous fungi M1, M2, M4 and M6, were isolated from this source and analyzed in this study.

Strains *Penicillium rubens* IF2SW-F4 and *Penicillium rubens* DSM 1075 were taken from the culture collection of the Aerospace Microbiology Research Group, German Aerospace Center (DLR). Strain IF2SW-F4 was originally isolated from a surface inside the International Space Station (ISS) during the NASA JPL Microbial Tracking studies [23]. DSM 1075, the corresponding type strain, was used as a reference in this study. CO_2_ levels on the ISS can be more than 20 times higher than in the earth’s atmosphere (0.03% at the time of publication), ranging from 0.3 to 0.7% [24]. For humans, these conditions can already have a medical impact in terms of increasingly reported headaches [25].

Filamentous fungi strains TS1 and TS2 were airborne and isolated from TSA (tryptic soy agar; Merck, Germany) and were included as reference strains from environments with non-elevated CO_2_ concentrations.

All strains listed above are yeasts and filamentous ascomycetes. Other taxa were not isolated from these samples.

### 2.2. Cultivation and Modified Atmosphere Packaging of Strains

Spore suspensions of all strains listed above were stored at −80 °C using Cryobank (Mast Group, Bootle, UK). For all subsequent experiments, the fungi were cultivated on YE agar (10.0 g glucose, 2.5 g yeast extract, 10 g bacteriological agar, 500 mL distilled H_2_O) or broth. All strains tested positive for growth at temperatures of 10 and 25 °C.

Growth under MA was assessed by placing inoculated YE agar Petri dishes or 50 mL inoculated YE broth in tissue culture flasks (Sarstedt, Nümbrecht Germany) in a polypropylene tray (ES-Plastic, Hutthurm, Germany), which was sealed with polyethylene foil (Südpack, Ochsenhausen, Germany) by a Multivac T200 traysealer (Multivac, Wolfertschwenden, Germany). A common gas composition for meat products, consisting of 80% O_2_ and 20% CO_2_, was used for all strains. The trays were incubated at 10 °C, to simulate the conditions of food storage, for the time span of four weeks, after which the growth was visually evaluated, and by measuring the optical density or glucose concentration in the media. In case the growth of each strain was sufficient (utilization of the glucose or an optical density above 0.1), biological triplicates were incubated under MAP for all subsequent experiments on agar for 14 days (yeasts) and in broth (filamentous fungi) until the remaining glucose concentration was approximately 25 mg/L (filamentous fungi). Concerning the yeasts, controls exposed to normal atmosphere and MAP samples were incubated at 10 °C for 14 days and at 25 °C for 3 days in order to distinguish CO_2_-induced effects on the membrane from temperature-related effects. In contrast, controls and MAP samples of the filamentous fungi were incubated at 10 °C, as well as samples at an elevated temperature of 25 °C and were harvested at growth phases as similar as possible by harvesting at 25 mg/L glucose. The gas compositions were immediately measured before harvesting. Trays with non-inoculated YE and trays without plates were also packed under MAP and incubated like the samples in order to determine how the gas composition differs due to CO_2_ diffusing into the agar and other factors changing the gas composition. The pH values of non-inoculated agar stored under MAP and the controls were measured after incubation.

### 2.3. Phylogenetic Analyses

The genomic DNA of the strains was extracted with a Qiagen DNeasy Blood and Tissue Kit (Qiagen, Hilden, Germany) and the supplementary protocol for yeasts using the enzyme lyticase or using a MasterPure Yeast DNA Purification kit (Lucigen, Biosearch Technologies, Hoddesdon, UK). The extraction was performed according to the instructions in the protocols, with an incubation time of 90 min after adding proteinase K using the Qiagen DNeasy Blood and Tissue Kit.

For the amplification of the 28S rRNA gene region or ITS region, the primer sets used were NL-1F (5′-GCATATCAATAAGCGGAGGAAAAG-3′) and NL-4R (5′-GGTCCGTGTTTCAAGACGG-3′) [26] for the sequencing of the D1/D2 region at the 5′ end of the large subunit 28S rRNA gene and ITS-1 (5′-TCCGTAGGTGAACCTGCGG-3′) and ITS-4 (5′-TCCTCCGCTTATTGATATGC-3′) for sequencing of the ITS region [27]. The PCR conditions were as follows: initial denaturation at 94 °C for 2 min; 36 cycles of denaturation at 94 °C for 0.5 min, annealing at 52 °C for 1 min, elongation at 72 °C for 1 min; final extension at 72 °C for 10 min, followed by a cooling phase at 4 °C for the 28S rRNA gene sequence and initial denaturation at 95 °C for 2 min; 35 cycles of denaturation at 95 °C for 0.3 min, annealing at 52 °C for 0.5 min, elongation at 72 °C for 1.5 min; final extension at 72 °C for 10 min, followed by a cooling phase at 4 °C for the ITS gene sequence. The PCR products were Sanger sequenced by Seqlab (Göttingen, Germany). Sequences were manually checked and edited with Chromas software (Version 2.6.6, Technelysium, South Brisbane, Australia). Partial sequences of other genes, namely ribosomal 18S (SSU) rRNA, *TEF1* (translation EF-1KA nucleotide sequence) and *RPB2* (RNA polymerase II subunit nucleotide sequence), were used for phylogenetic analyses of strain WT5. Amplification and sequencing of the 18S rRNA, RPB2 and TEF1 genes were performed with the following primer pairs: NS1 and NS8, RPB2-7cR and RPB2-5F, YTEF-1G and YTEF-6G, respectively [28,29,30,31]. Nucleotide sequences were assembled with Sequencher software (Version 5.4.6, GeneCodes Inc., Ann Arbor, MI, USA).

The gene sequences were compared with publicly available sequences from GenBank using BLAST version 2.12.0 (Basic local alignment search tool, National Centre for Biotechnological Information, Bethesda, MD, USA; [32]) and the MycoID (www.mycobank.org, accessed on 26 September 2023) database. Models and environmental samples were excluded in the search. The results of the sequencing and BLAST search are shown in Table 1.

For strain WT5, nucleotide sequence alignments were aligned in the dataset previously used by Kachalkin et al. [33] in the description and placement of *Zygotorulaspora dagestanica* using the online version of the MAFFT algorithm [34]. Phylogenetic relationships in the concatenated alignment of the five loci were inferred by the Maximum-Likelihood (ML) method with RAxML GUI 2.0 [35] with 100 thorough bootstrap replicates. A model test tool implemented in the software was used to determine the best substitution model for each partition, the general time reversible model GTR + G + I (SSU and LSU), the Kimura model K80 + G (5.8S rRNA gene) and transition models TIM2 (*RPB2*) and TIM3 (*TEF1*). Single-gene best ML trees were combined into a single file and analyzed with Splitstree 4.10 [36] using the ConsensusNetwork (threshold 0.1; edges weight sum of nodes) algorithm. The approach has been previously used to visualize and delimit closely related species in the *Papiliotrema flavescens* species complex [37] and species in the family *Saccharomycetaceae* [38]. The dataset for the multigene phylogenetic tree consisted of 48 taxa and that for the phylogenetic network was reduced to 46 taxa for the phylogenetic tree analysis to include taxa for which all sequences of the five gene regions were available. The alignment included representatives of the family *Saccharomycetaceae*, the WGD clade (the clade characterized by the whole-genome duplication) in the whole-genome analysis of ascomycetous yeasts by Shen et al. [39], as well as outgroup taxa from the families *Debaryomycetaceae*, *Phaffomycetaceae* and *Pichiaceae*. The resulting alignment consisted of 3597 characters: 1654 in the nearly complete 18S rRNA gene (SSU), 584 in D1/D2 domains of the 26S/28S rRNA gene (LSU), 160 in 5.8S rRNA gene, 448 in *TEF1* and 751 in *RPB2*.

### 2.4. Fatty Acid Analysis

For the analysis of fungal fatty acids, the samples were prepared in the form of fatty acid methyl esters [40] from 40 mg of yeast cell material grown on agar plates or from 20 mg of lyophilized fungal cell material. The methyl esters were chromatographically separated via the GC-MSD system 8890 (Agilent Technologies, Santa Clara, CA, USA) with a 5% phenyl methyl silicone capillary column (0.25 mm by 30 m) and identified as previously described [41]. Samples that contained both the oleic (C_18:1_ cis 9) and linolenic acid (C_18:3_ cis 9, 12, 15) were additionally analyzed using a GC 2010 plus system (Shimadzu, Duisburg, Germany) with a flame ionization detector [42] and a Phenomenex ZX-WaxPlus column (0.25 mm by 30 m) to discriminate these two compounds. After relatively quantifying the samples, the degree of unsaturation (DU) within the fatty acid profiles was calculated using Formula 1. The analyzed fatty acids include data sets from growth at 10 °C with MAP treatment (20% CO_2_, 80% O_2_), controls with growth at 10 °C without MA and growth at 25 °C in regular atmosphere. All fatty acid profiles are listed in Table 2 (yeasts) and Table 3 (filamentous fungi), alongside the calculated average melting temperature (WAMT) of each organisms’ fatty acid profile and the DU. WAMT values were calculated according to Seel (2018) [43].

Formula (1): Formula for calculating the degree of unsaturation (DU) within the extracted fatty acids extracted from the fungal strains.

(1) $$DU=1\times \%\frac{monoenes}{100}+2\times \%\frac{dienes}{100}+3\times \%\frac{trienes}{100}$$

### 2.5. Physiology and Chemotaxonomy of Strains WT5 and WR1

Growth tests for strains WT5 and WR1 were performed in liquid media according to the methods described by Kurtzman et al. [44]. A Microscope Nikon ECLIPSE Ni-E equipped with phase-contrast and digital interference optics and a digital camera DS-Ri2 were used for microscopy. Polar lipid patterns of membrane lipids were extracted and analyzed for the strains WT5 and WR1 using two-dimensional thin-layer chromatography, as described previously [45]. For polar lipid extraction, the yeasts were cultivated in YE broth until the solution reached an optical density of 1.0 ± 0.2 at 625 nm. Furthermore, the fatty acids of the two yeast strains were extracted and analyzed as described above. The results are displayed in Table 2.

## 3. Results

### 3.1. Growth Assessment

The growth of all strains used in this study decreased under MAP, except for the two yeast strains *Candida sake* HT4 and *P. fermentans* WT2, which grew as well as in the control cultures under normal atmosphere. A general observation in light of consistency was that the yeast appearance on the CO_2_-incubated plates was generally more mucous than that of the controls. The mycelial clumps in liquid culture were observed to be smaller and denser under MA in most of the cases. Furthermore, in terms of pigmentation, differences were noticed between cultures grown under elevated CO_2_ concentrations and controls. Untypical orange to pink pigmentation was observed in the CO_2_ samples of *C. sake* HT4, *P. fermentans* WT2, strains WT5 and WR1, as well as *Candida oleophila* WT7, while the controls were generally white to beige in color. In filamentous fungi, the most apparent pigmentation change was noted in *P. rubens* DSM 1075, which developed a pink color under CO_2_ (from originally white), and *Penicillium griseofulvum* HR2, *P. rubens* IF2SW-F4, *Penicillium tardochrysogenum* TS1 and *Penicillium griseofulvum* TS2, which equally changed from a white and beige to a yellowish to orange color. The formation of exudates, which has been observed in *P. rubens* IF2SW-F4, *P. griseofulvum* HR2 and especially *P. glandicola* M6 under normal atmospheres on solid medium, was strongly reduced under elevated CO_2_ conditions.

The pH value of the agar was lowered due to carbonic acid formation after MAP, leading to a decrease from 7.0 ± 0.1 under normal atmosphere at 10 °C to a pH of 6.1 ± 0.1 under increased CO_2_ levels.

### 3.2. Phylogenetic Analyses

**Figure 1:**
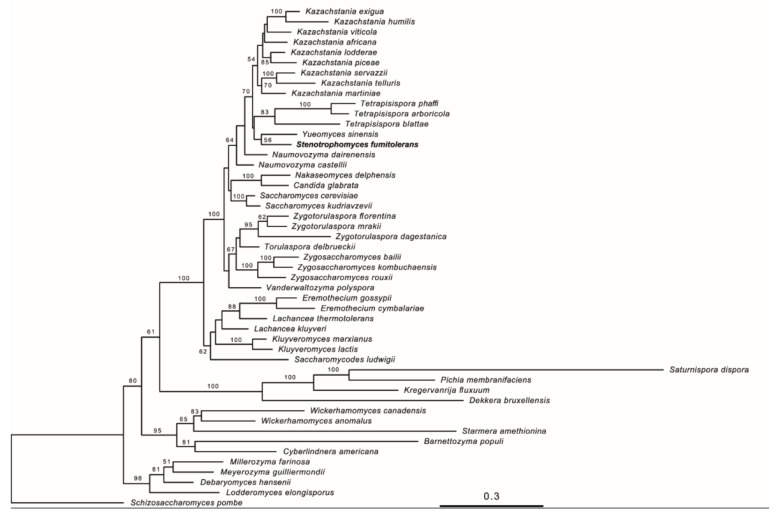
Maximum likelihood tree of *Stenotrophomyces fumitolerans* WT5 sp. nov. and related species and genera of the family *Saccharomycetaceae* with outgroup taxa from families *Debaryomycetaceae*, *Phaffomycetaceae* and *Pichiaceae* obtained from the combined analysis of SSU, 5.8S rRNA, LSU, TEF1 and RPB2 genes. The tree is rooted with sequences of *Schizosaccharomyces pombe*. The numbers provided on branches are frequencies (>50%) with which a given branch appeared in 100 through bootstrap replications. The scale bars indicate the numbers of expected substitutions accumulated per site. Accession numbers of nucleotide sequences are provided in Supplementary Table S1.

The multi-locus phylogenetic analysis located the yeast strain WT5 in a well-supported (bootstrap value of 100% for the ML tree) clade composed of genera *Kazachstania*, *Naumovozyma*, *Saccharomyces*, *Nakaseomyces*, *Yueomyces*, *Tetrapisispora*, *Torulaspora*, *Zygotorulaspora*, *Zygosaccharomyces* and *Vanderwaltozyma* (Figure 1). The sub-clade containing genera *Kazachstania*, *Yueomyces*, *Tetrapisispora* and the novel yeast received moderate (bootstrap value of 70% for the ML tree) support in the analysis. The phylogenetic analysis was consistent with previous analyses by Kachalkin et al. [33] and Shen et al. [39]. The potential new yeast species was placed in the phylogenetic tree close to the genera *Tetrapisispora* and *Yueomyces*, although the support for the placement was weak (Figure 1). The main clade of the genus *Tetrapisispora* received very good (ML: 100%) support and *Tetrapisispora blattae* clustered as the next species with good (ML: 83%) support. The phylogenomic analysis by Shen et al. [39] demonstrated the polyphyly of the genus *Tetrapisispora* and the placement of the species *Tetrapisispora blattae* close to *Yueomyces sinensis*. In the same analysis, *Vanderwaltozyma polyspora* was clustered with other *Tetrapisispora* species.

**Figure 2:**
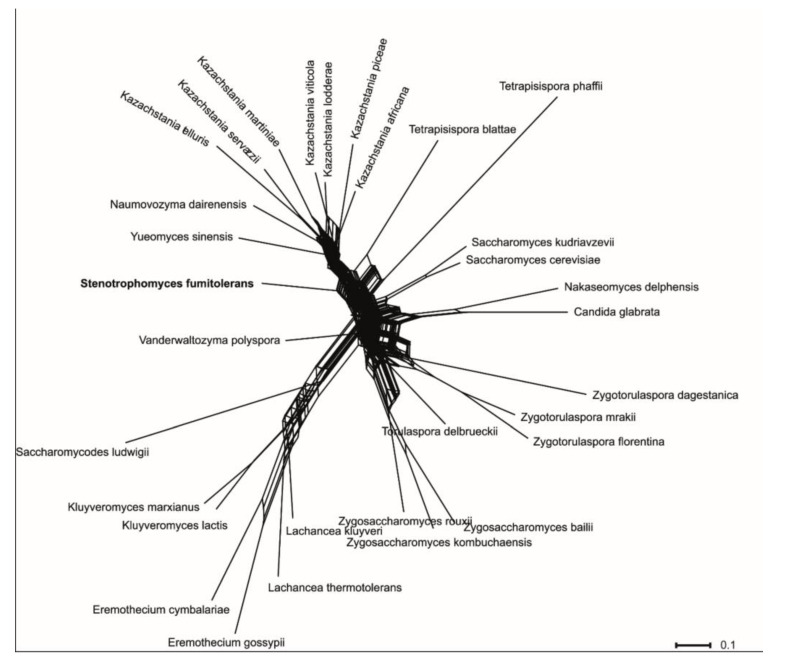
Consensus network obtained from the combined analyses of single-gene maximum likelihood trees of SSU, 5.8S rRNA, LSU, TEF1 and RPB2 alignments. The scale indicates mean distance obtained from the analysis of single-gene trees. Accession numbers of nucleotide sequences are provided in Supplementary Table S1.

The network analyses revealed a complex structure of the clade and showed discrepancies between single-gene datasets (Figure 2). In agreement with previous results, the genus *Kazachstania* displayed high genetic heterogeneity and shared ancestry with *Naumovozyma* (see also [39]). Compared to other genera, *Tetrapisispora blattae* was rather distant from the type species of the genus *Tetrapisispora phaffii*, corroborating the results of the previous phylogenomic study [39]. The strain WT5 was placed in the network between *Yueomyces sinensis* and *Vanderwaltozyma polyspora*. Taking into consideration relative position and distances between taxa in the phylogenetic tree and network analyses and the phylogenomic analysis of Shen et al. [39], it is very unlikely that the novel yeast species will build a good phylogenetic cluster either with *Tetrapisispora phaffii*, *Tetrapisispora blattae*, *Yueomyces sinensis* or *Vanderwaltozyma polyspora*. The new yeast is as distant from the aforementioned taxa as the members of different genera comprising the WGD clade [39].

### 3.3. Fatty Acid Compositions

**Figure 3:**
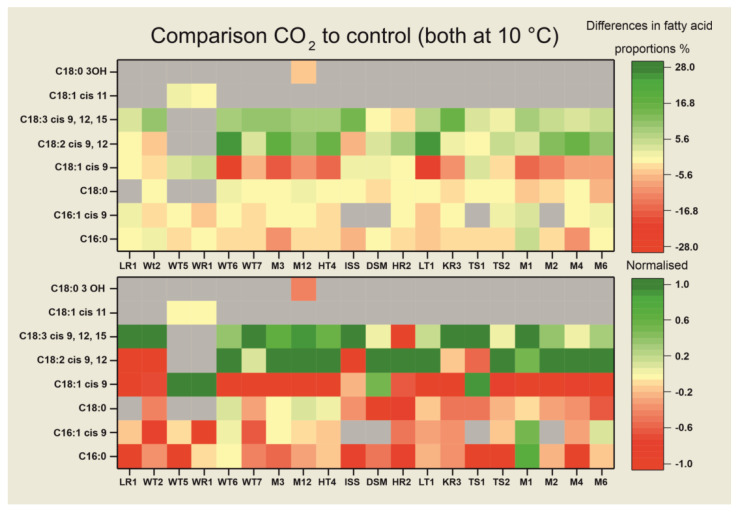
Comparison of averaged changes in total fatty acid compositions of MAP-treated samples at 10 °C to the controls at 10 °C. Increases in fatty acid contents of MAP-treated samples compared to the controls are highlighted in green, while decreases are highlighted in red with the intensity of the color correlating to the extent of the difference. In the lower graph, differences in the fatty acid contents are normalized in order to individually display changes for each sample. When a fatty acid is not detected in a sample or the content is below <1%, it is not included, and is therefore displayed in grey. The organisms are mentioned by their strain designations. “DSM” resembles the strain DSM 1075, and “ISS” resembles the strain IF2SW-F4.

The fatty acid composition of the fungi analyzed in this work mainly consisted of palmitic and stearic acids, as well as their unsaturated derivatives. *Stenotrophomyces fumitolerans* WT5 and WR1 both lacked polyunsaturated linoleic and linolenic acids. *R. oryzae* M1 was the only species with γ- instead of α-linolenic acid, which has been reported to be a characteristic of *Zygomycetes* [46]. Figure 3 displays the differences between the MAP-treated samples and the controls (at 10 °C, with natural atmospheric composition) as a heatmap. The WAMT values of the CO_2_-treated samples were lower in comparison to the controls in every case–except for *S. fumitolerans* WR1–meaning that the organisms increasingly synthesize fatty acids with lower melting temperatures, which fluidizes the membranes. This is mainly due to the elevated DU, which was increased for all samples treated with increased CO_2_ levels. The effect of carbon dioxide on the WAMT values and the DU was much higher than the effect from the temperature shift from 25 to 10 °C.

As can be seen in the upper graph of Figure 3, *Apiotrichum gracile* WT6, *Didymella corylicola* LT1, *Rhodotorula alborubescens* M3 and *C. sake* HT4 show the strongest adaptation to CO_2_-enriched atmosphere at 10 °C incubation temperature in terms of changed fatty acid incorporation. Changes in the WAMT values and proportions of fatty acids are displayed in Table 2 and Table 3. The organisms *Candida zeylanoides* LR1, *P. rubens* DSM 1075 and *S. fumitolerans* WR1, on the other hand, do not show any significant changes (*p* > 0.05). Moreover, *S. fumitolerans* WT5, *P. tardochrysogenum* TS1 and *P. griseofulvum* TS2 only have one significant change (*p* < 0.05), namely a slightly decreased proportion of C_16:0_ under increased CO_2_ concentrations. In the lower graph, displaying the normalized values, it is noticeable that under CO_2_ the proportion of C_18:3_ cis 9, 12, 15 (or C_18:3_ cis 6, 9, 12 in the case of *R. oryzae* M1) is increased in almost all species or remains the same, whereas it is only decreased in *P. griseofulvum* HR2. The same applies for C_18:2_ cis 9, 12, which is increased in most species, but decreased in *C. zeylanoides* LR1, *P. fermentans* WT2, *P. rubens* IF2SW-F4, *N. tetraspora* KR3 and *P. tardochrysogenum* TS1. Furthermore, most species have a decreased content of C_18:1_ cis 9 under elevated CO_2_ levels, except for *S. fumitolerans* WT5 and WR1, where it increased as the only unsaturated fatty acid present, which was also observed for *P. rubens* DSM1075 and *P. tardochrysogenum* TS1. The contents of C_16:0_, C_16:1_ cis 9 and C_18:0_ remained the same or were reduced under CO_2_, except for *R. oryzae* M1, where C_16:0_ and C_16:1_ cis 9 increased under CO_2_.

As for the changes in fatty acids after incubation at increased temperatures of 25 °C, the total DU is lower, compared to the strains cultivated at 10 °C with carbon dioxide treatment and to the controls. This consequently led to an elevated WAMT value for those samples (Table 2 and Table 3). The most significant changes occur in *A. gracile* WT6, *P. rubens* DSM1075, *P. glandicola* M6, *Alternaria* sp. M4 and *D. prosopidis* LT1. The main differences between the fatty acids of fungi grown at 10 °C and at 25 °C are the decrease in linolenic acid and the increase in linoleic acid at 25 °C. Moreover, the content of the palmitoleic acid is decreased, particularly in yeasts. The contents of oleic and palmitic acid, on the other hand, are highest at the elevated temperature and lowest at CO_2_ treatment.

### 3.4. Taxonomy of Novel Yeast Species

The sequence analyses of strains WT5 and WR1 revealed that these strains represent a new species. Physiologically, the new yeast species is characterized by a narrow spectrum of utilized carbon and nitrogen compounds and shares this similarity with members of the genus *Tetrapisispora* and *Yueomyces*, with *Yueomyces sinensis* being the only known species of the genus. The new species ferments and assimilates glucose and galactose, and can additionally grow on trehalose (and also ferments it) and glycerol, which makes it a potential food spoilage microorganism. The new yeast shares these physiological properties with *Tetrapisispora namnaonensis* and differs from this species in the ability to grow in the presence of 50% glucose (negative for *T. namnaonensis*) and 10% NaCl (negative for strains WT5 and WR1). The yeast is homothallic and produces unconjugated autogamic or pedogamic persistent asci; it does not produce true hyphae. The neighboring genera *Tetrapisispora*, *Yueomyces* and *Vanderwaltozyma* are characterized by a remarkable diversity of morphological features of sexual reproduction (the formation of asci and ascospore numbers). Neither growth characteristics nor sexual reproduction can be used to convincingly support the placement of this yeast in either of these genera. Taking into account the position of the new yeast in multi-locus phylogenetic analyses, the physiological profile and culture characteristics, we decided to propose a new genus for these yeast strains rather than to place them in any of the already existing genera. We advocate that it is better to propose a new genus to accommodate this single-species lineage than to substantially increase phylogenetic heterogeneity of the neighboring genera, be that *Tetrapisispora*, *Yueomyces* or *Vanderwaltozyma*.

The polar lipid pattern consisted of phosphatidylethanolamine, dimethyl-phosphatidylethanolamine, phosphatidylcholine and phosphatidylinositol. The fatty acid profiles of strains WT5 and WR1 were similar under all conditions, including MAP treatment and different incubation temperatures. At 25 °C, the main fatty acids of these organisms consisted of C_16:1_ cis 9 (65.5 to 66.4%), C_18:1_ cis 9 (17.2 to 18.2%) and C_16:0_ (9.3 to 10.5%). At 10 °C the amount of C_16:1_ cis 9 was increased to 74.5 to 79.7%, whereas both C_18:1_ fatty acids were lowered. Exposition to elevated amounts of CO_2_ did not have a strong effect on the fatty acid profile in contrast to the temperature change. Polyunsaturated fatty acids were missing, which was only described for a few other members of the family *Saccharomycetaceae*, such as *Saccharomyces cerevisiae* [47], as well as for other yeast genera such as *Hanseniaspora* and *Schizosaccharomyces* [48].

The strains WT5 and WR1 were deposited at the DSMZ (Deutsche Sammlung von Mikroorganismen und Zellkulturen) under the strain numbers DSM 113852 and DSM 113853, respectively.

### 3.5. Description of Stenotrophomyces fumitolerans Gen. Nov., Sp. Nov.

***Stenotrophomyces***, D. Heidler von Heilborn, J. Reinmüller, A. Yurkov, P. Stehle, R. Möller, A. Lipski, gen. nov. MB# 849905

**Ethymology:** The genus name refers to the narrow assimilation spectrum of carbon sources.

**Description:** Cells are ellipsoid, ovoid or elongate and reproduce by multilateral budding. Asci with one or two spherical ascospores. Narrow assimilation spectrum of carbon sources. Glucose and galactose are fermented. Growth in vitamin-free medium. No starch-like substance is produced. Urea hydrolysis and the Diazonium blue B reaction are negative.

**Type species: *Stenotrophomyces fumitolerans***

***Stenotrophomyces fumitolerans***, D. Heidler von Heilborn, J. Reinmüller, A. Yurkov, P. Stehle, R. Möller, A. Lipski, sp. nov. MB# 849906

**Ethymology:** The species name refers to the ability of the species to tolerate CO_2_-rich environments.

**Figure 4:**
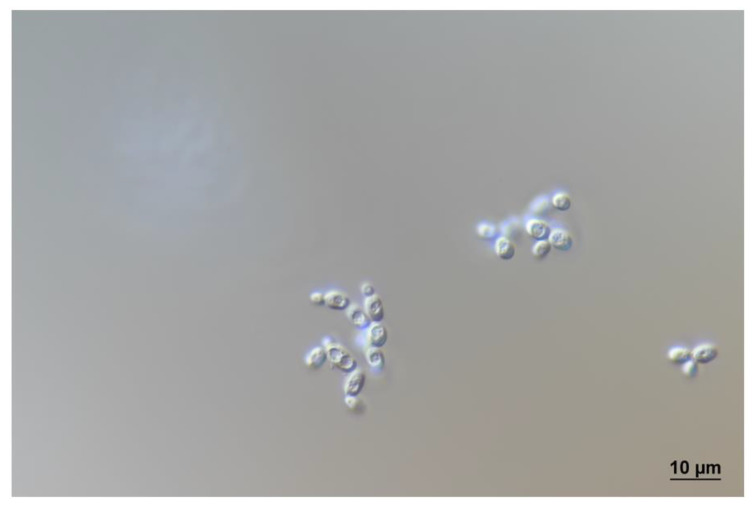
*Stenotrophomyces fumitolerans* WT5 cells and asci on minimal medium after 7 days at 22 °C obtained with DIC optic.

On Glucose Peptone Yeast Extract Agar (GPYA) and 5% malt extract agar (MEA), after 7 days at 22 °C, the streak is cream-colored, butyrous and smooth, with an entire margin. The profile is flat. Cells are ovoid to ellipsoid, 2.5–6.0 × 2.0–4.0 μm, singly occur and in pairs (Figure 4). Pseudohyphae and true hyphae are not formed on GPYA, MEA, potato dextrose agar or corn meal agar. Growth on minimal medium without amino acids. Vegetative reproduction is by multilateral budding.

Ascospores are produced by both strains on GPYA, MEA and on minimal medium without amino acids after 7–14 days at 22 °C (Figure 4). Asci are persistent, often unconjugated or arise following conjugation between a cell and its bud. One or two spheroid (diameter of 2.5–3.0 μm) ascospores are formed per ascus.

Fermentation of d-glucose, d-galactose and trehalose is positive. Glucose, d-galactose, trehalose and glycerol are assimilated. No growth occurs on l-sorbose, d-glucosamine, d-ribose, d-xylose, l-arabinose, d-arabinose, sucrose, maltose, methyl α-d-glucoside, cellobiose, salicin, melibiose, lactose, raffinose, melezitose, inulin, soluble starch, erythritol, ribitol, l-arabinitol, d-glucitol, d-mannitol, galactitol, myo-inositol, 2-keto-d-gluconate, 5-keto-d-gluconate, d-gluconate, d-glucoronate, d-galacturonate, dl-lactic acid, succinic acid, citric acid, methanol or ethanol. Nitrogen compounds: ammonium sulfate is assimilated, but not potassium nitrate, sodium nitrite, ethylamine, l-lysine, cadaverine, creatine, creatinine, glucosamine, imidazole or d-tryptophan. Growth in the presence of 5% (*w*/*v*) NaCl and 50% (*w*/*v*) glucose is positive. Weak growth in the presence of 8% (*w*/*v*) NaCl, and no growth in the presence of 10% (*w*/*v*) NaCl and 60% (*w*/*v*) glucose. Growth in vitamin-free medium positive. No starch-like substance is produced. Urea hydrolysis and the Diazonium blue B reaction are negative. Maximum growth temperature is 28 °C. The minimum growth temperature is 2 °C.

The holotype, strain WT5, was isolated from a vegetarian wrap in June 2020 in Bonn (Germany). It is preserved in a metabolically inactive state in the German Collection of Microorganisms and Cell Cultures, Braunschweig, Germany, under the number DSM 113852. The isotype is preserved in a metabolically inactive state in the CBS culture collection of the Westerdijk Fungal Biodiversity Institute (Utrecht, the Netherlands) under the number CBS 18379 and in the HAMBI microbial culture collection, Helsinki, Finland, under the number HAMBI 3777.

Other strains studied: paratype WR1 (DSM 113853, HAMBI 3776) from the same source.

## 4. Discussion

Elevated concentrations of carbon dioxide cause a reduction in microbial and, in particular, fungal growth, which is technically used in MAP within the food industry [7,8]. The inhibition mechanism is not fully understood yet, but it is believed to be a complex interaction of several effects, including membrane-, ion- and pH-related effects [13]. These, for example, comprise a disorder of the phospholipids within the cell membrane caused by the CO_2_ molecules [49], as well as a decrease in extra- and intracellular pH, leading to an inhibition of microbial growth and elevated energy consumption to maintain pH homeostasis [13,50]. Due to its ability to solve in water and fat [51], it is very likely that the inhibition mechanism of carbon dioxide includes the phospholipid bilayer of the membrane as a target. This can be assessed by analyzing the fatty acid composition. We assumed that CO_2_ stress would lead to a fluidization of the membrane to which the organisms react by incorporation of fatty acids with a higher melting temperature, leading to a solidification of the membrane and an increase of the WAMT. Other fat-soluble stressors have already been found to have an impact on the polar lipid and fatty acid compositions of microorganisms by disturbing the lipid layer, such as the amphiphilic ethanol [52]. It was shown in *S. cerevisiae* that its ethanol tolerance is highly dependent on the oleic acid content [53].

We found that carbon dioxide stress resulted in a significant elevation of the DU of fatty acids in yeasts and filamentous fungi, mainly due to the increased incorporation of linolenic acid. The WAMT did slightly decrease, meaning that no solidification of the membrane was observed, but a fluidization of the membrane. On the contrary, increased temperatures of 25 °C led to an increase of the WAMT and a decrease in the DU, indicating a solidification of the membrane in order to adapt to the elevated temperature.

In this study, several strains from natural habitats (mofettes), carbon dioxide including MA-packed food, the ISS and airborne fungi as controls were used to study commonly used adaptation mechanisms to elevated CO_2_ levels. In addition to the elevated carbon dioxide levels, temperature adjustments were tested as comparative parameters, where fatty acid modifications served as a reference to assess membrane fluidization effects. We found that an MA of 80% O_2_ and 20% CO_2_ led to reduced fungal biomass production, reduced sporulation and reduced exudate formation on agar compared to the controls grown under normal atmosphere. The mycelial clumps often formed in liquid cultures were smaller and denser under MA. This could be due to the growth-inhibitory effects of CO_2_. In a study by Fairclough et al., 20% CO_2_ MA fungistatically acted and negatively affected the growth of *Penicillium roqueforti* and the pigmentation of immature conidiospores; the effects were reversible and the pigmentation returned in the normal atmosphere [54]. The same effects of altered coloration have been observed in the present study. An MA consisting of 80% CO_2_ and 20% O_2_ has been reported to exert lethal effects after incubation for 60 days on *Xeromyces bisporus* and *Eurotium chevalieri* [55], but we did not observe any lethal effects in our experiments, which were performed with lower CO_2_ concentrations.

We have demonstrated that artificial atmospheres with elevated CO_2_ levels induce changes in the fungal fatty acid profiles. The most pronounced changes in the majority of fungi used were the higher proportions of linolenic and linoleic acids and the decreased levels of oleic acid. Consequently, these effects were displayed by the DU in the total fatty acid content, which was increased in all strains. Because of the rather low melting temperatures of the polyunsaturated fatty acids, the WAMT values were lower under elevated CO_2_, indicating fluidization of the membrane. The strains did not respond to the presumed fluidizing effect of the CO_2_ by solidification of their membrane through increasing the WAMT. We assume that the physiological reaction observed may be one reason, in these concentrations, for the non-lethal but growth-limiting effect of carbon dioxide. Literature on the effects of high concentrations of CO_2_ on microbial fatty acids, especially on fungal fatty acids, is scarce. However, the high degree of unsaturation under CO_2_, which was observed in this work, is consistent with the results for *S. cerevisiae* by Castelli et al. [56]. The group found that elevated CO_2_ concentrations, as well as elevated HCO_3_^-^ concentrations, lead to a higher degree of unsaturation in fatty acids and that with increasing CO_2_ concentrations, regardless of the pH value, the total amount of fatty acids and lipids increases.

In contrast, meat-spoilage bacteria and green algae showed a decreased unsaturation degree due to the incorporation of mainly saturated fatty acids under 30% CO_2_ and 70% O_2_ in bacteria [57] and under 2% CO_2_ in algae [58]. For the green algae, a total increase in fatty acids in high CO_2_-treated cells was observed as well [58]. Available studies were mostly performed with very few isolates and did not cover a reasonable range of taxa. It has been furthermore suggested that the observed inhibitory effects of CO_2_ may also depend on other factors, such as the incubation temperature, growth medium, water activity and pH value [59].

The exact functions of specific fatty acids in fungi have not been fully elucidated yet. Linoleic acid and its derivatives have been shown to increase conidial development in *Aspergillus* and asexual spore production, and might act as signaling molecules modulating fungal sporulation [60]. The chemical mechanism underlying the effect is the formation of oxylipins from linoleic acids or their derivatives, by the incorporation of molecular oxygen with the help of oxygenases. The enzyme activities of stearoyl-CoA Δ9 and oleoyl-CoA Δ12 desaturases have been studied in dependence of the growth stage and temperature in *Yarrowia lipolytica* [61]. At 10 °C, the maximum activity of stearoyl-CoA desaturase was half of that at 25 °C, while the oleoyl-CoA desaturase activity showed the opposite trend, which is consistent with the observed higher unsaturation at lower temperatures. In the proposed main pathway of oleic and linoleic acids in *Y. lipolytica*, linoleic acid is synthesized by the Δ12 desaturase from oleic acid on phosphatidylcholine as a carrier, while oleic acid has been formed from 18:0-CoA by the Δ9 desaturase and then by acyl exchange onto phosphatidylcholine [61].

Concerning effects on human nutrition, polyunsaturated fatty acids have been reported to have a positive impact, including cholesterol-lowering effects, leading to a reduced risk of cardiovascular diseases [62]. If the DU in yeasts and fungi is increased after incubation in a CO_2_-containing MA or after CO_2_ treatment in general, this modification of the fungal lipid profile could be used in perspective when cultivating edible fungi to increase their nutritional value.

The temperature-dependent adaptation mechanisms through the change of fatty acid compositions have been found to vary between different fungal species [63] and are of interest for this study in terms of comparing membrane adaptations to temperature shifts with those to increased CO_2_ concentrations. The adaptation strategy, referred to as homeoviscous adaptation, was initially reported for the bacterium *Escherichia coli*, which favored fatty acids with higher melting points when incubated at higher temperatures in order to maintain membrane fluidity [64]. This strategy was also observed in 14 out of the 20 organisms investigated in the present study, the WAMT values of which were lower when incubated at a temperature of 10 °C, mainly due to the increased amount of linolenic acid and decreased concentration of palmitic acid. Cold stress adaptation is reportedly achieved by an increased DU and a decrease in the average chain length of fatty acids [65] by the induction of fatty acid desaturases and dehydrases, which decrease the proportion of saturated fatty acids, and (less frequently) decrease the chain length [66]. Similar observations were made for the fungus *Pleurotus ostreatus*, where lowered temperatures of 12 °C resulted in an increase in fatty acid unsaturation in comparison to the controls at 27 °C, mainly by an increase in linoleic acid [16]. In the present work, the temperature change from 25 °C to 10 °C caused similar changes, namely the increased ratio of polyunsaturated to monounsaturated fatty acids. Therefore, organisms that are better adapted to cold environments have been shown to produce a higher proportion of unsaturated lipids that result in lower WAMT values–an effect that was furthermore described for the amphiphilic ethanol [53], and which we unexpectedly observed for carbon dioxide stress in this study as well. Initially, we assumed that fungi would respond to high levels of membrane-soluble CO_2_, similar to their heat adaptation, by solidification of the membrane. Some organisms showed similar fatty acid profiles when grown under elevated CO_2_ and under normal atmosphere at 25 °C, including *A. gracile* WT6 and *D. corylicola* LT1, both of which showed strong adaptation to elevated CO_2_ and to the elevated temperature compared to the controls at 10 °C.

We found that the increase in temperature, which results in an increased WAMT for the majority of strains, does not have the same adaptation effect on fatty acid compositions as the effect of compounds that solve in water and in lipids, such as carbon dioxide, which we used in our study, or ethanol, as described in [53].

In addition, similar adaptation mechanisms were found irrespective of the origin of the strains, including mofettes, MAP food products, the ISS, with elevated CO_2_ concentrations, or the airborne strains with regular atmospheric CO_2_ exposition. This indicates that the increase in the unsaturation of fatty acids is a general response of yeasts and filamentous fungi to carbon-dioxide-induced stress and is different from the stress-response induced by increased temperatures.

The isolation of different strains from environments with elevated CO_2_ concentrations furthermore resulted in the discovery of a new yeast genus and species proposed as *Stenotrophomyces fumitolerans* gen. nov., sp. nov. Investigating such extreme environments and the adapted organisms could be of great importance concerning new opportunities in biotechnology, such as pigments, enzymes or secondary metabolites [67]. In the case of MA-packed food, these organisms can also play an important role as potential food spoilage organisms, especially when they grow at low temperatures, such as *Stenotrophomyces fumitolerans* WT5, which demonstrates growth down to 2 °C. As mentioned above, the production of polyunsaturated fatty acids in fungi under carbon dioxide-rich conditions might be used to improve the nutritional value of food products.

## Figures and Tables

**Table 1:** Isolation source and identification of the different fungal strains using ITS and/or 28S rRNA gene regions according to GenBank. Sequences of isolates were only compared to type strain sequences. Models and environmental samples were excluded. All strains were sequenced during this project, with the exception of the two strains IF2SW-F4 and DSM 1075.

Source	Strain	Species According to NCBI BLAST	Sequence Similarity with Closely-Related Type Strain	Sequence Accession No.
Vegetarian wrap	WR1	*Torulaspora globosa*	96.9% ‡ (KY109865.1)	OQ255941
	WT2	*Pichia fermentans*	99.8% ‡ (MK394169.1)	OQ255937
	WT5	*Vanderwaltozyma verrucispora*	89.0% * (NR_137559.1)	OQ255923
		*Torulaspora globosa*	96.9% ‡ (KY109865.1)	OQ255942
		Further accession numbers for SSU-ITS-LSU fragment, RPB2 and TEF1	-	OQ708375, OQ715316, OR661267
	WT6	*Apiotrichum gracile*	99.8% ‡ (KY106124.1)	OQ255938
	WT7	*Candida oleophila*	99.6% ‡ (NG_060820.1)	OQ255939
Minced Meat	HR2	*Penicillium griseofulvum*	99.8% * (MH854925.1)	OQ255924
		Penicillium dipodomyus †	100% ‡ (MH874450.1)	OQ255931
	HT4	*Candida sake*	100% ‡ (KY106745.1)	OQ255932
Salmon	LR1	*Candida zeylanoides*	100% ‡ (NG_060834.1)	OQ255933
	LT1	*Didymella corylicola* †	100% ‡ (MN954290.1)	OQ255934
Coffee	KR3	*Neurospora tetraspora*	98.9% ‡ (NG_068996.1)	OQ255940
Airborne	TS1	*Penicillium tardochrysogenum* †	100% * (MH865983.1)	OQ255929
		*Penicillium dipodomyus* †	100% ‡ (MH874450.1)	OQ255935
	TS2	*Penicillium griseofulvum*	99.8% * (MH854925.1)	OQ255930
		*Penicillium tardochrysogenum* †	100% ‡ (NG_070021.1)	OQ255936
Mofettes	M1	*Rhizopus oryzae*	100% * (DQ641279.1)	OQ255925
	M2	*Cladosporium subuliforme* †	100% * (MH864124.1)	OQ255922
	M3	*Rhodotorula alborubescens*	99.6% * (NR_153197.1)	OQ255926
	M4	*Alternaria alstroemeriae* †	100% * (NR_163686.1)	OQ255927
	M6	*Penicillium glandicola*	100% * (MH860946.1)	OQ255928
	M12	*Rhodotorula babjevae*	99.4% * (NR_077096.1)	OQ504373
International Space Station (ISS)	IF2SW-F4	*Penicillium rubens*	100.0% (MT558923.1)	JACSPE000000000
Cantaloupe melon	DSM 1075	*Penicillium rubens*	100% * (NR_111815.1)	PKG00000000, as published before [22]

* ITS nucleotide sequence; ‡ LSU nucleotide sequence; † Additionally shares 100% sequence similarity with other strains.

**Table 2:** Fatty acid composition of the examined yeasts under elevated CO_2_ levels (20% CO_2_) at 10 °C, and under normal atmospheres at 10 °C and 25 °C. The values represent the average of three biological replicates (n = 3) and the standard deviation in % of the total area of all fatty acids in the sample. WAMT resembles the weighted average melting temperature of the membrane in °C. DU shows the degree of unsaturation. nd, fatty acids not detected in the replicates.

Organisms	Growth at	C14:0	C16:0	C16:1 cis 9	C18:0	C18:1 cis 9	C18:2 cis 9, 12	C18:3 cis 9, 12, 15	C18:1 cis 11	C18:0 3OH	WAMT (°C)	DU
*Candida zeylanoides* LR1	10 °C, CO_2_		10.4 ± 0.5	8.9 ± 1.8	<1	38.7 ± 3.1	24.8 ± 1.9	17.3 ± 2.6	nd		7.7 ± 0.2	1.49
	10 °C	<1	11.5 ± 1.5	8.4 ± 2.1		39.6 ± 3.1	25.8 ± 0.5	14.7 ± 4.0		nd	8.7 ± <0.1	1.44
	25 °C		15.1 ± 0.6	4.5 ± 0.3		41.3 ± 1.7	27.0 ±0.3	12.0 ± 1.3			11.5 ± 0.1	1.36
*Pichia fermentans* WT2	10 °C, CO_2_		11.4 ± 1.6	19.8 ± 1.7	0.5 ± 0.2	22.9 ± 2.1	22.9 ± 1.3	22.4 ± 0.4	nd		6.4 ± 0.7	1.56
	10 °C	<1	11.1 ± 0.9	23.1 ± 0.4	1.0 ± 0.1	24.9 ± 1.2	27.1 ± 1.0	12.8 ± 0.5		nd	7.6 ± 0.5	1.41
	25 °C		14.8 ± 0.4	15.7 ± 1.6	2.2 ± 0.3	21.4 ± 1.3	35.6 ± 0.2	10.3 ± 0.3			9.9 ± 0.6	1.39
*Stenotrophomyces fumitolerans* WT5	10 °C, CO_2_		5.6 ± 0.7	74.2 ± 2.6	<1	16.4 ± 2.0	nd	nd	3.8 ± 0.7		7.0 ± 0.7	0.94
	10 °C	<1	8.3 ± 0.3	74.5 ± 0.8		13.5 ± 0.3			3.8 ± 0.7	nd	8.4 ± 0.2	0.92
	25 °C		9.3 ± 1.5	65.5 ± 3.3		18.2 ± 1.9			7.0 ± 1.0		10.0 ± 1.0	0.91
*Stenotrophomyces fumitolerans* WR1	10 °C, CO_2_		8.6 ± 0.5	75.6 ± 0.1	<1	13.1 ± 0.2	nd	nd	2.7 ± 0.3		8.3 ± 0.3	0.91
	10 °C	<1	9.0 ± 0.5	79.7 ± 4.6		9.2 ± 3.5			2.9 ± 0.8	nd	8.2 ± 0.2	0.91
	25 °C		10.5 ± 0.4	66.4 ± 1.2		17.2 ± 1.0			5.9 ± 0.4		10.4 ± 0.4	0.89
*Apiotrichum gracile* WT6	10 °C, CO_2_		16.2 ± 1.0	1.4 ± 0.3	2.4 ± 2.8	19.6 ± 1.0	47.2 ± 4.2	14.0 ± 1.7	nd		8.7 ± 2.3	1.57
	10 °C	<1	19.1 ± 1.8	2.3 ± 0.4	2.3 ± 0.4	47.5 ± 3.0	22.9 ± 3.0	5.9 ± 1.4		nd	17.2 ± 2.0	1.13
	25 °C		20.8 ± 1.1	0.8 ± 0.3	3.9 ± 1.2	24.0 ± 1.7	46.4 ± 2.8	4.2 ± 3.9			14.9 ± 0.6	1.30
*Candida oleophila* WT7	10 °C, CO_2_		12.4 ± 0.5	12.4 ± 1.1	0.3 ± 0.1	35.6 ± 0.4	19.1 ± 0.9	20.1 ± 0.3	nd		8.9 ± 0.3	1.37
	10 °C	<1	14.3 ± 0.5	15.7 ± 1.0	0.8 ± 0.1	41.9 ± 3.8	16.8 ± 1.3	10.3 ± 2.7		nd	12.6 ± 1.1	1.22
	25 °C		14.7 ± 0.4	1.8 ± 0.1	3.7 ± 0.3	47.8 ± 0.7	23.7 ± 0.4	8.2 ± 0.8			15.2 ± 0.6	1.22
*Candida sake* HT4	10 °C, CO_2_		11.1 ± 0.5	11.5 ± 2.7	0.7 ± 0.3	34.0 ± 8.8	28.7 ± 6.8	14.0 ± 4.5	nd		8.2 ± 0.2	1.45
	10 °C	<1	14.3 ± 1.2	14.7 ± 0.1	1.1 ± 0.1	50.5 ± 3.4	13.7 ± 3.1	5.8 ± 1.6		nd	14.6 ± 1.6	1.10
	25 °C		12.3 ± 0.7	10.5 ± 0.2	1.5 ± 0.3	48.0 ± 4.6	23.0 ± 3.1	4.7 ± 1.3			12.8 ± 0.8	1.19
*Rhodotorula alborubescens* M3	10 °C, CO_2_		10.4 ± 0.5	0.9 ± 0.1	1.9 ± 0.1	28.5 ± 0.8	39.1 ± 1.1	19.2 ± 0.4	nd		6.4 ± 0.4	1.65
	10 °C	<1	20.4 ± 1.3	1.4 ± 0.2	2.4 ± 0.5	46.8 ± 2.3	20.9 ± 2.1	8.1 ± 1.4		nd	17.9 ± 1.5	1.14
	25 °C		18.1 ± 0.5	0.4 ± 0.1	4.0 ± 0.2	54.3 ± 0.8	17.6 ± 0.6	5.6 ± 0.4			19.0 ± 0.3	1.06
*Rhodotorula babjevae* M12	10 °C, CO_2_	1.0 ± <0.1	13.7 ± 0.3	2.1 ± 0.2	1.4 ± 0.2	25.8 ± 0.9	25.6 ± 0.2	18.9 ± 0.3		11.5 ± 0.5	19.2 ± 0.4	1.70
	10 °C	1.0 ± 0.2	16.9 ± 0.9	3.2 ± 0.5	1.0 ± 0.4	36.5 ± 2.8	15.5 ± 0.8	9.6 ± 2.3	nd	16.1 ± 1.3	28.7 ± 0.8	1.48
	25 °C	1.1 ± 0.1	17.7 ± 1.2	1.2 ± 0.1	1.7 ± 0.2	39.0 ± 3.3	20.2 ± 2.9	10.3 ± 1.9		8.5 ± 0.5	22.1 ± 1.7	1.38

**Table 3:** Fatty acid composition of the examined filamentous fungi under elevated CO_2_ levels (20% CO_2_) at 10 °C, and under normal atmospheres at 10 °C and 25 °C. The values represent the average of three biological replicates (n = 3; except for M2 10 °C, where n = 2) and the standard deviation in % of the total area of all fatty acids in the sample. WAMT resembles the weighted average melting temperature of the membrane in °C. DU shows the degree of unsaturation. * *R. oryzae* M1 has C18:3 cis 6, 9, 12 instead of C18:3 cis 9, 12, 15.

Organisms	Growth at	C16:0	C16:1 cis 9	C18:0	C18:1 cis 9	C18:2 cis 9, 12	C18:3 cis 9, 12, 15	WAMT (°C)	DU
*Penicillium rubens* IF2SW-F4	10 °C, CO_2_	8.8 ± 0.5		6.5 ± 1.0	11.1 ± 1.7	43.7 ± 3.8	30.0 ± 4.8	4.7 ± 0.2	1.89
	10 °C	14.9 ± 0.6	<1	7.2 ± 1.4	10.2 ± 1.2	51.0 ± 2.7	16.7 ± 0.6	10.0 ± 1.7	1.64
	25 °C	16.1 ± 1.1		3.6 ± 0.9	8.7 ± 0.5	60.0 ± 2.7	11.6 ± 1.0	8.0 ± 1.3	1.63
*Penicillium rubens* DSM 1075	10 °C, CO_2_	8.9 ± 0.4		6.8 ± 2.9	4.2 ± 2.2	47.3 ± 4.6	32.8 ± 4.5	3.6 ± 1.4	1.97
	10 °C	10.6 ± 1.3	<1	10.1 ± 2.9	2.7 ± 0.9	44.5 ± 6.2	33.0 ± 5.6	6.8 ± 1.5	1.91
	25 °C	16.0 ± 1.6		4.0 ± 0.6	3.5 ± 0.5	61.5 ± 5.2	15.1 ± 3.2	7.0 ± 1.6	1.72
*Penicillium griseofulvum* HR2	10 °C, CO_2_	10.5 ± 1.2	1.6 ± 0.5	2.3 ± 1.1	6.9 ± 1.0	64.2 ± 3.3	14.5 ± 2.3	2.7 ± 1.8	1.80
	10 °C	13.0 ± 0.4	1.6 ± 0.3	4.2 ± 0.8	8.0 ± 0.9	55.7 ± 3.3	17.5 ± 2.2	6.0 ± 0.5	1.74
	25 °C	14.8 ± 0.9	0.8 ± 0.1	2.9 ± 0.3	8.0 ± 0.4	70.2 ± 2.2	3.4 ± 1.1	6.8 ± 0.8	1.59
*Didymella corylicola* LT1	10 °C, CO_2_	14.8 ± 2.3	3.9 ± 0.7	1.5 ± 0.2	12.9 ± 2.2	58.6 ± 2.3	8.4 ± 2.6	6.8 ± 2.2	1.59
	10 °C	18.8 ± 1.5	8.1 ± 2.2	3.0 ± 1.0	33.8 ± 3.9	33.8 ± 5.0	2.5 ± 0.5	15.5 ± 2.0	1.17
	25 °C	14.4 ± 1.8	1.0 ± 0.6	2.8 ± 0.1	20.7 ± 5.4	57.9 ± 6.5	3.2 ± 1.5	9.1 ± 2.4	1.47
*Neurospora tetraspora* KR3	10 °C, CO_2_	10.2 ± 1.8	1.5 ± 1.0	1.5 ± 0.4	5.8 ± 0.6	54.3 ±2.4	26.8 ± 3.5	1.1 ± 1.7	1.96
	10 °C	12.1 ± 1.3	3.2 ± 0.8	4.7 ± 1.8	15.5 ± 3.2	53.2 ± 1.6	11.2 ± 1.3	7.7 ± 1.6	1.59
	25 °C	15.0 ± 1.2	1.5 ± 0.1	8.0 ± 2.8	25.7 ± 3.0	45.0 ± 3.1	4.8 ± 2.2	14.4 ± 2.1	1.32
*Penicillium tardochrysogenum* TS1	10 °C, CO_2_	11.0 ± 1.1	<1	4.5 ± 0.4	13.4 ± 1.5	42.5 ± 4.2	28.5 ± 6.5	5.4 ± 1.1	1.84
	10 °C	13.8 ± 0.9		5.8 ± 0.9	10.7 ± 1.3	44.0 ± 2.6	25.6 ± 3.3	7.9 ± 0.4	1.76
	25 °C	15.0 ± 0.4		2.5 ± 0.4	23.3 ± 3.0	50.4 ± 6.9	8.8 ± 3.3	9.4 ± 0.9	1.50
*Penicillium griseofulvum* TS2	10 °C, CO_2_	10.3 ± 0.8	1.7 ± 0.9	2.5 ± 1.1	6.2 ± 1.4	64.6 ± 4.8	14.7 ± 2.3	2.6 ± 1.5	1.81
	10 °C	12.9 ± 0.6	1.6 ± 0.4	2.6 ± 0.8	8.1 ± 0.8	60.8 ± 3.5	14.0 ± 4.2	4.9 ± 0.6	1.73
	25 °C	14.6 ± 1.0	0.8 ± 0.1	3.0 ± 0.6	8.6 ± 0.7	70.2 ± 3.0	2.7 ± 0.9	6.9 ± 1.2	1.58
*Rhizopus oryzae* M1 *	10 °C, CO_2_	25.5 ± 4.4	8.0 ± 2.9	2.1 ± 0.3	35.1 ± 4.1	11.6 ± 2.0	17.7 ± 4.8	19.2 ± 4.0	1.19
	10 °C	20.3 ± 0.4	5.0 ± 1.0	6.5 ± 2.5	50.4 ± 1.6	8.8 ± 2.4	9.0 ± 2.0	22.0 ± 2.3	1.00
	25 °C	22.0 ± 3.5	0.9 ± 0.4	6.1 ± 1.1	42.1 ± 1.5	19.4 ± 1.5	9.5 ± 1.4	20.9 ± 2.8	1.10
*Cladosporium subuliforme* M2	10 °C, CO_2_	20.6 ± 2.2	<1	2.4 ± 0.4	16.2 ± 0.9	49.9 ± 2.1	10.9 ± 0.8	11.7 ± 1.5	1.49
	10 °C	22.8 ±1.6		5.4 ± 2.8	28.2 ± 0.2	37.3 ± 0.1	6.3 ± 0.8	18.1 ± 1.0	1.22
	25 °C	29.1 ± 2.4		8.4 ± 1.8	33.7 ± 0.8	27.9 ± 4.0	0.9 ± 0.2	26.1 ± 2.7	0.92
*Alternaria alstroemeriae* M4	10 °C, CO_2_	14.6 ± 1.5	0.9 ± <0.1	5.0 ± 1.0	8.3 ± 1.6	63.7 ± 1.3	7.5 ± 2.0	8.2 ± 1.7	1.59
	10 °C	24.1 ± 1.3	1.3 ± <0.1	6.2 ± 1.1	16.9 ± 0.6	47.6 ± 3.2	4.0 ± 0.2	17.5 ± 1.9	1.25
	25 °C	22.3 ± 2.2	1.5 ± 0.1	6.2 ± 0.6	33.9 ± 2.3	35.8 ± 0.6	0.3 ± <0.1	20.0 ± 1.6	1.08
*Penicillium glandicola* M6	CO_2_	12.0 ± 0.7	2.8 ± 0.3	2.4 ± 0.4	7.7 ± 1.3	61.3 ± 2.0	13.9 ± 2.7	4.1 ± 0.6	1.75
	10 °C	12.9 ± 0.6	1.2 ± 0.1	8.3 ± 0.2	17.0 ± 1.7	50.6 ± 0.9	10.0 ± 2.5	11.2 ± 0.8	1.49
	25 °C	16.4 ± 1.4	1.4 ± 0.1	6.9 ± 1.4	36.7 ± 2.1	37.3 ± 1.5	1.4 ± 0.5	16.9 ± 1.8	1.17

## Data Availability

The datasets generated during and/or analyzed during the current study are provided in this manuscript.

## Supplementary Materials

The following supporting information can be downloaded at: https://www.mdpi.com/article/10.3390/jof9101031/s1, Table S1: List of the novel species *Stenotrophomyces fumitolerans* WT5, the representatives of the family *Saccharomycetaceae* and outgroup taxa from families *Debaryomycetaceae*, *Phaffomycetaceae*, and *Pichiaceae* used for multi-locus phylogenetic analysis. Type strains were used for the analysis.

## Abbreviations

DLR: German Aerospace Center; DSMZ, Deutsche Sammlung von Mikroorganismen und Zellkulturen; DU, degree of unsaturation; ISS, International Space Station; MA, Modified Atmosphere; MAP, Modified Atmosphere Packaging/packed; ML, Maximum-Likelihood; ppm, parts per million; WAMT, weighted average melting temperature.

## References

[1] Ropars J., Giraud T. (2022). Convergence in domesticated fungi used for cheese and dry-cured meat maturation: Beneficial traits, genomic mechanisms, and degeneration. Curr. Opin. Microbiol..

[2] Kure C.F., Skaar I. (2019). The fungal problem in cheese industry. Curr. Opin. Food Sci..

[3] Lambert A.D., Smith J.P., Dodds K.L. (1991). Shelf life extension and microbiological safety of fresh meat—A review. Food Microbiol..

[4] Kurtzman C.P. (2015). Identification of food and beverage spoilage yeasts from DNA sequence analyses. Int. J. Food Microbiol..

[5] Pitt J.I., Hocking A.D. (2022). Fungi and Food Spoilage.

[6] Fröhlich-Nowoisky J., Pickersgill D.A., Després V.R., Pöschl U. (2009). High diversity of fungi in air particulate matter. Proc. Natl. Acad. Sci. USA.

[7] Mullan M., McDowell D., Coles R., Kirwan M. (2011). Modified Atmosphere Packaging. Food and Beverage Packaging Technology.

[8] Taniwaki M.H., Hocking A.D., Pitt J.I., Fleet G.H. (2009). Growth and mycotoxin production by food spoilage fungi under high carbon dioxide and low oxygen atmospheres. Int. J. Food Microbiol..

[9] Taniwaki M., Hocking A., Pitt J., Fleet G. (2001). Growth of fungi and mycotoxin production on cheese under modified atmospheres. Int. J. Food Microbiol..

[10] Gonzalez-Fandos E., Maya N., Martínez-Laorden A., Perez-Arnedo I. (2020). Efficacy of Lactic Acid and Modified Atmosphere Packaging against Campylobacter jejuni on Chicken during Refrigerated Storage. Foods.

[11] Santillan E.-F.U., Shanahan T.M., Omelon C.R., Major J.R., Bennett P.C. (2015). Isolation and characterization of a CO_2_-tolerant Lactobacillus strain from Crystal Geyser, Utah, U.S.A. Front. Earth Sci..

[12] Šibanc N., Zalar P., Schroers H.-J., Zajc J., Pontes A., Sampaio J.P., Maček I. (2018). *Occultifur mephitis* f.a., sp. nov. and other yeast species from hypoxic and elevated CO_2_ mofette environments. Int. J. Syst. Evol. Microbiol..

[13] Garcia-Gonzalez L., Geeraerd A.H., Spilimbergo S., Elst K., Van Ginneken L., Debevere J., Van Impe J., Devlieghere F. (2007). High pressure carbon dioxide inactivation of microorganisms in foods: The past, the present and the future. Int. J. Food Microbiol..

[14] Isenschmid A., Marison I.W., von Stockar U. (1995). The influence of pressure and temperature of compressed CO_2_ on the survival of yeast cells. J. Biotechnol..

[15] Beccaccioli M., Reverberi M., Scala V. (2019). Fungal lipids: Biosynthesis and signalling during plant-pathogen interaction. Front. Biosci. (Landmark Ed).

[16] Pedneault K., Angers P., Avis T.J., Gosselin A., Tweddell R.J. (2007). Fatty acid profiles of polar and non-polar lipids of *Pleurotus ostreatus* and *P. cornucopiae* var. ‘citrino-pileatus’ grown at different temperatures. Mycol. Res..

[17] Suutari M. (1995). Effect of growth temperature on lipid fatty acids of four fungi (*Aspergillus niger*, *Neurospora crassa*, *Penicillium chrysogenum*, and *Trichoderma reesei*). Arch. Microbiol..

[18] Sumner J.L., Morgan E.D., Evans H.C. (1969). The effect of growth temperature on the fatty acid composition of fungi in the order Mucorales. Can. J. Microbiol..

[19] Weete J.D. (1980). Lipid Biochemistry of Fungi and Other Organisms.

[20] Phillips R.L., Zak D.R., Holmes W.E., White D.C. (2002). Microbial community composition and function beneath temperate trees exposed to elevated atmospheric carbon dioxide and ozone. Oecologia.

[21] Lipson D.A., Wilson R.F., Oechel W.C. (2005). Effects of elevated atmospheric CO_2_ on soil microbial biomass, activity, and diversity in a chaparral ecosystem. Appl. Environ. Microbiol..

[22] Wang L., Groenewald M., Wang Q.-M., Boekhout T. (2015). Reclassification of Saccharomycodes sinensis, Proposal of Yueomyces sinensis gen. nov., comb. nov. within Saccharomycetaceae (Saccharomycetales, Saccharomycotina). PLoS ONE.

[23] Simpson A.C., Urbaniak C., Bateh J.R., Singh N.K., Wood J.M., Debieu M., O’hara N.B., Houbraken J., Mason C.E., Venkateswaran K. (2021). Draft Genome Sequences of Fungi Isolated from the International Space Station during the Microbial Tracking-2 Experiment. Microbiol. Resour. Announc..

[24] James J.T. (2007). The Headache of Carbon Dioxide Exposures.

[25] Law J., Van Baalen M., Foy M., Mason S.S., Mendez C., Wear M.L., Meyers V.E., Alexander D. (2014). Relationship between carbon dioxide levels and reported headaches on the international space station. J. Occup. Environ. Med..

[26] Kwiatkowski N.P., Babiker W.M., Merz W.G., Carroll K.C., Zhang S.X. (2012). Evaluation of nucleic acid sequencing of the D1/D2 region of the large subunit of the 28S rDNA and the internal transcribed spacer region using SmartGene IDNS corrected software for identification of filamentous fungi in a clinical laboratory. J. Mol. Diagn..

[27] White T.J., Bruns T., Lee S., Taylor J. (1990). Amplification and direct sequencing of fungal ribosomal RNA genes for phylogenetics. PCR Protocols.

[28] Kurtzman C.P., Robnett C.J. (2003). Phylogenetic relationships among yeasts of the ‘Saccharomyces complex’ determined from multigene sequence analyses. FEMS Yeast Res..

[29] Liu Y.J., Whelen S., Hall B.D. (1999). Phylogenetic relationships among ascomycetes: Evidence from an RNA polymerse II subunit. Mol. Biol. Evol..

[30] Matheny P.B., Liu Y.J., Ammirati J.F., Hall B.D. (2002). Using RPB1 sequences to improve phylogenetic inference among mushrooms (Inocybe, Agaricales). Am. J. Bot..

[31] Rehner S.A., Buckley E. (2005). A Beauveria phylogeny inferred from nuclear ITS and EF1-alpha sequences: Evidence for cryptic diversification and links to Cordyceps teleomorphs. Mycologia.

[32] Altschul S.F., Gish W., Miller W., Myers E.W., Lipman D.J. (1990). Basic local alignment search tool. J. Mol. Biol..

[33] Kachalkin A.V., Abdullabekova D.A., Magomedova E.S., Yurkov A.M. (2021). *Zygotorulaspora dagestanica* sp. nov., a novel ascomycetous yeast species associated with the Georgian honeysuckle (*Lonicera iberica* M. Bieb.). Int. J. Syst. Evol. Microbiol..

[34] Katoh K., Rozewicki J., Yamada K.D. (2019). MAFFT online service: Multiple sequence alignment, interactive sequence choice and visualization. Brief. Bioinform..

[35] Edler D., Klein J., Antonelli A., Silvestro D. (2021). raxmlGUI 2.0: A graphical interface and toolkit for phylogenetic analyses using RAxML. Methods Ecol. Evol..

[36] Huson D.H., Bryant D. (2006). Application of phylogenetic networks in evolutionary studies. Mol. Biol. Evol..

[37] Yurkov A., Guerreiro M.A., Sharma L., Carvalho C., Fonseca Á. (2015). Multigene assessment of the species boundaries and sexual status of the basidiomycetous yeasts *Cryptococcus flavescens* and *C. terrestris* (Tremellales). PLoS ONE.

[38] Wu Q., James S.A., Roberts I.N., Moulton V., Huber K.T. (2008). Exploring contradictory phylogenetic relationships in yeasts. FEMS Yeast Res..

[39] Shen X.-X., Opulente D.A., Kominek J., Zhou X., Steenwyk J.L., Buh K.V., Haase M.A., Wisecaver J.H., Wang M., Doering D.T. (2018). Tempo and Mode of Genome Evolution in the Budding Yeast Subphylum. Cell.

[40] Sasser M. (1990). Identification of Bacteria by Gas Chromatography of Cellular Fatty Acids.

[41] Lipski A., Altendorf K. (1997). Identification of Heterotrophic Bacteria Isolated from Ammonia-supplied Experimental Biofilters. Syst. Appl. Microbiol..

[42] van Lent D.M., Egert S., Wolfsgruber S., Kleineidam L., Weinhold L., Wagner-Thelen H., Maier W., Jessen F., Ramirez A., Schmid M. (2021). Eicosapentaenoic Acid Is Associated with Decreased Incidence of Alzheimer’s Dementia in the Oldest Old. Nutrients.

[43] Seel W., Flegler A., Zunabovic-Pichler M., Lipski A. (2018). Increased Isoprenoid Quinone Concentration Modulates Membrane Fluidity in Listeria monocytogenes at Low Growth Temperatures. J. Bacteriol..

[44] Kurtzman C.P., Fell J.W., Boekhout T., Robert V. (2011). Methods for Isolation, Phenotypic Characterization and Maintenance of Yeasts. The Yeasts.

[45] von Heilborn D.H., Reinmüller J., Hölzl G., Meier-Kolthoff J.P., Woehle C., Marek M., Hüttel B., Lipski A. (2021). *Sphingomonas aliaeris* sp. nov., a new species isolated from pork steak packed under modified atmosphere. Int. J. Syst. Evol. Microbiol..

[46] Lösel D.M. (1990). Lipids in the Structure and Function of Fungal Membranes.

[47] Timke M., Wang-Lieu N.Q., Altendorf K., Lipski A. (2008). Identity, beer spoiling and biofilm forming potential of yeasts from beer bottling plant associated biofilms. Antonie Van Leeuwenhoek.

[48] Loureiro V. (1999). The prevalence and control of spoilage yeasts in foods and beverages. Trends Food Sci. Technol..

[49] Jones R.P., Greenfield P.F. (1982). Effect of carbon dioxide on yeast growth and fermentation. Enzym. Microb. Technol..

[50] Hutkins R.W., Nannen N.L. (1993). pH Homeostasis in Lactic Acid Bacteria. J. Dairy Sci..

[51] Münch M., Guillard V., Gaucel S., Destercke S., Thévenot J., Buche P. (2023). Composition-based statistical model for predicting CO_2_ solubility in modified atmosphere packaging application. J. Food Eng..

[52] Tesnière C. (2019). Importance and role of lipids in wine yeast fermentation. Appl. Microbiol. Biotechnol..

[53] You K.M., Rosenfield C.-L., Knipple D.C. (2003). Ethanol tolerance in the yeast Saccharomyces cerevisiae is dependent on cellular oleic acid content. Appl. Environ. Microbiol..

[54] Fairclough A.C., Cliffe D.E., Knapper S. (2011). Factors affecting *Penicillium roquefortii* (*Penicillium glaucum*) in internally mould ripened cheeses: Implications for pre-packed blue cheeses. Int. J. Food Sci. Technol..

[55] Taniwaki M.H., Hocking A.D., Pitt J.I., Fleet G.H. (2010). Growth and mycotoxin production by fungi in atmospheres containing 80% carbon dioxide and 20% oxygen. Int. J. Food Microbiol..

[56] Castelli A., Littarru G.P., Barbaresi G. (1969). Effect of pH and CO_2_ concentration changes on lipids and fatty acids of Saccharomyces cerevisiae. Arch. Mikrobiol..

[57] Kolbeck S., Kienberger H., Kleigrewe K., Hilgarth M., Vogel R.F. (2021). Effect of high levels of CO_2_ and O_2_ on membrane fatty acid profile and membrane physiology of meat spoilage bacteria. Eur. Food Res. Technol..

[58] Tsuzuki M., Ohnuma E., Sato N., Takaku T., Kawaguchi A. (1990). Effects of CO(2) Concentration during Growth on Fatty Acid Composition in Microalgae. Plant Physiol..

[59] Farber J.M. (1991). Microbiological Aspects of Modified-Atmosphere Packaging Technology—A Review 1. J. Food Prot..

[60] Calvo A.M., Hinze L.L., Gardner H.W., Keller N.P. (1999). Sporogenic effect of polyunsaturated fatty acids on development of *Aspergillus* spp. Appl. Environ. Microbiol..

[61] Ferrante G., Kates M. (1983). Pathways for desaturation of oleoyl chains in *Candida lipolytica*. Can. J. Biochem. Cell Biol..

[62] Kris-Etherton P.M., Hecker K.D., Binkoski A.E. (2004). Polyunsaturated Fatty Acids and Cardiovascular Health. Nut. Rev..

[63] Stahl P.D., Klug M.J. (1996). Characterization and differentiation of filamentous fungi based on Fatty Acid composition. Appl. Environ. Microbiol..

[64] Sinensky M. (1974). Homeoviscous adaptation—A homeostatic process that regulates the viscosity of membrane lipids in Escherichia coli. Proc. Natl. Acad. Sci. USA.

[65] Krell T. (2018). Cellular Ecophysiology of Microbe: Hydrocarbon and Lipid Interactions.

[66] Al-Fageeh M.B., Smales C.M. (2006). Control and regulation of the cellular responses to cold shock: The responses in yeast and mammalian systems. Biochem. J..

[67] Buzzini P., Turchetti B., Yurkov A. (2018). Extremophilic yeasts: The toughest yeasts around?. Yeast.

